# Supplementary material for: Occurrence and transmission potential of asymptomatic and presymptomatic SARS-CoV-2 infections: A living systematic review and meta-analysis
Source: PLoS Med. 2020 Sep 22;17(9):e1003346. doi: 10.1371/journal.pmed.1003346 (PMC7508369; doi:10.1371/journal.pmed.1003346)
Supplement: S1 Text — (DOCX) [file pmed.1003346.s002.docx]

S1 Text. Search strings

## From: Living Evidence on COVID-19 (https://ispmbern.github.io/covid-19/living- [review/collectingdata.html,](https://ispmbern.github.io/covid-19/living-review/collectingdata.html) accessed 10.06.2020)

We retrieve data from [EMBASE](http://www.embase.com/) via OVID, [MEDLINE](https://www.ncbi.nlm.nih.gov/pubmed/) via PubMed, BioRxiv and MedRxiv.

**Search terms**

When searches are updated, references that are identified that were not in the database before, are inserted by date (**date_entrez**) they were indexed in remote database, the date they are inserted in OUR database is formatted as the ‘**strategydate**’ (raw data is available [here](https://github.com/ZikaProject/COVID_references)).

**01.05.2020**

EMBASE:

(SARS coronavirus/ or middle east respiratory syndrome/ or severe acute respiratory syndrome/ or (coronavirus* or corona virus* or HCoV* or ncov* or covid or covid19 or sars-cov* or sarscov* or Sars-coronavirus* or Severe Acute Respiratory Syndrome Coronavirus*).mp.) and 20191201:20301231.(dc).

# 29.04.2020

## MEDLINE:

("coronavirus"[MH] OR "coronavirus infections"[MH] OR "coronavirus"[TW] OR "corona virus"[TW] OR "HCoV"[TW] OR "nCov"[TW] OR "covid"[TW] OR "covid19"[TW] OR "Severe Acute Respiratory Syndrome Coronavirus 2"[TW] OR "SARS-CoV2"[TW] OR "SARS-CoV 2"[TW] OR "SARS Coronavirus 2"[TW] OR "MERS- CoV"[TW]) AND (2019/1/1:3000[PDAT])

# 01.04.2020

## From 01.04.2020, we retrieve the currate BioRxiv/MedRxiv dataset [Link](https://connect.medrxiv.org/relate/content/181)

**26.03.2020**

MEDLINE:

("Wuhan coronavirus" [Supplementary Concept] OR "COVID-19" OR SARS-CoV-2 OR "2019 ncov"[tiab] OR (("novel coronavirus"[tiab] OR "new coronavirus"[tiab]) AND (wuhan[tiab] OR 2019[tiab])) OR 2019-nCoV[All Fields] OR (wuhan[tiab] AND coronavirus[tiab]))

## EMBASE:

(nCoV or 2019-nCoV or ((new or novel or wuhan) adj3 coronavirus) or covid19 or covid-19 or SARS-CoV-2).mp.

## BioRxiv/MedRxiv:

ncov or corona or wuhan or COVID or SARS-CoV-2

## With the kind support of the [Public Health & Primary Care Library PHC,](https://www.unibe.ch/university/services/university_library/faculty_libraries/medicine/public_health_amp_primary_care_library_phc/index_eng.html) and following guidance of the [Medical Library Association](https://www.mlanet.org/p/cm/ld/fid%3D1713)

**01.01.2020**

MEDLINE:

("Wuhan coronavirus" [Supplementary Concept] OR "COVID-19" OR "2019 ncov"[tiab] OR (("novel coronavirus"[tiab] OR "new coronavirus"[tiab]) AND (wuhan[tiab] OR 2019[tiab])) OR 2019-nCoV[All Fields] OR (wuhan[tiab] AND coronavirus[tiab])))))

## EMBASE:

ncov OR (wuhan AND corona) OR COVID

## BioRxiv/MedRxiv:

ncov or corona or wuhan or COVID

## We retained publications that used the keywords listed below in the title or abstract.

"asymp*" OR "pre-symp*" OR "presymp*" OR "preclinical" OR "pre-clinical" OR "without symptoms" OR "no symptoms" OR "free of symptoms" OR "non-symp*" OR "nonsymp*" OR "symptom-free" OR "symptomfree"
