# Supplementary material for: Occurrence and transmission potential of asymptomatic and presymptomatic SARS-CoV-2 infections: A living systematic review and meta-analysis
Source: PLoS Med. 2020 Sep 22;17(9):e1003346. doi: 10.1371/journal.pmed.1003346 (PMC7508369; doi:10.1371/journal.pmed.1003346)
Supplement: S1 Fig — (PDF) [file pmed.1003346.s003.pdf]

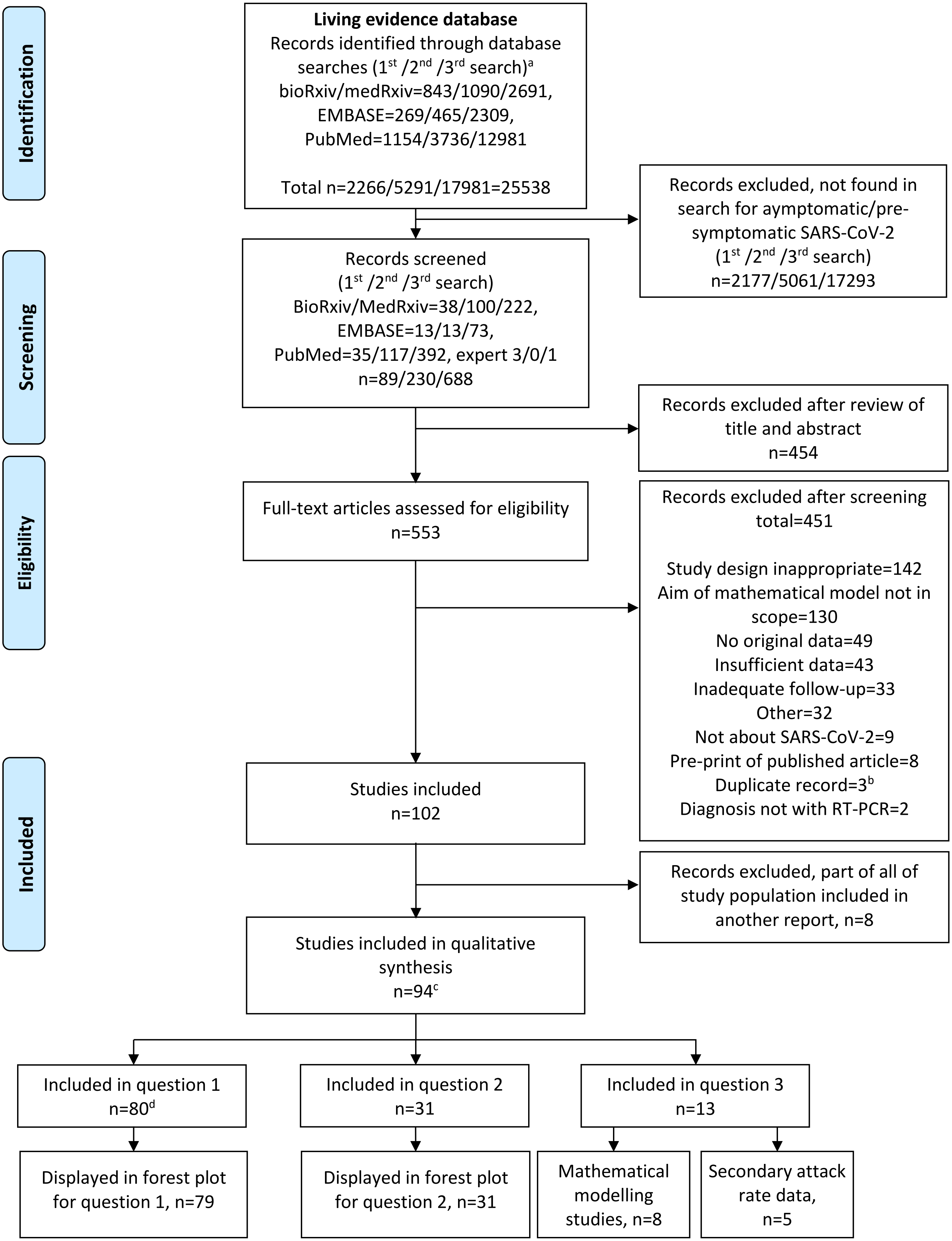

Figure S1. Flow chart of identified, excluded and included records as of 10 June 2020

[a] Numbers of new records at each search: 1st search, 25.03.2020; 2nd search, 20.04.2020; 3rd search, 10.06.2020

[b] Duplicate records are identical articles in different databases, found after automated de-duplication

[c] 11 studies were included in the first review and 37 studies were included in the first update

[d] Study by Mizumoto et al. in qualitative synthesis but not included in forest plot
