# Supplementary material for: Occurrence and transmission potential of asymptomatic and presymptomatic SARS-CoV-2 infections: A living systematic review and meta-analysis
Source: PLoS Med. 2020 Sep 22;17(9):e1003346. doi: 10.1371/journal.pmed.1003346 (PMC7508369; doi:10.1371/journal.pmed.1003346)
Supplement: S2 Fig — (PDF) [file pmed.1003346.s004.pdf]

Study	Events	Total	Prop.	95% CI
Tong, ZD [cluster:1]	1	2	0.50	[0.01;0.99]
Hoehl, S	1	2	0.50	[0.01;0.99]
Huang, R	1	2	0.50	[0.01;0.99]
Jiang, XL [cluster:2]	1	2	0.50	[0.01;0.99]
Jiang, XL [cluster:3]	1	2	0.50	[0.01;0.99]
Jiang, X	1	3	0.33	[0.01;0.91]
Liao, J [cluster:2]	1	3	0.33	[0.01;0.91]
Tong, ZD [cluster:2]	2	3	0.67	[0.09;0.99]
Hu, Z	1	4	0.25	[0.01;0.81]
Luo, SH	1	4	0.25	[0.01;0.81]
See, KC	1	4	0.25	[0.01;0.81]
Jiang, XL [cluster:1]	1	4	0.25	[0.01;0.81]
Liao, J [cluster:3]	1	4	0.25	[0.01;0.81]
Chan, JF	1	5	0.20	[0.01;0.72]
Liao, J [cluster:1]	1	5	0.20	[0.01;0.72]
Ye, F	1	5	0.20	[0.01;0.72]
Bai, Y	1	6	0.17	[0.00;0.64]
Luo, Y	5	6	0.83	[0.36;1.00]
Pongpirul, WA	1	11	0.09	[0.00;0.41]
Hijnen, D	1	11	0.09	[0.00;0.41]
Danis, K	1	13	0.08	[0.00;0.36]
Böhmer, MM	1	16	0.06	[0.00;0.30]
Zou, L	1	18	0.06	[0.00;0.27]
Chang, L	2	4	0.50	[0.07;0.93]
Zhang, J	2	5	0.40	[0.05;0.85]
Zhang, B	2	7	0.29	[0.04;0.71]
Huang, L	2	7	0.29	[0.04;0.71]
Qian, G	2	8	0.25	[0.03;0.65]
Roxby, AC	3	6	0.50	[0.12;0.88]
Yang, N	2	10	0.20	[0.03;0.56]
Tan, YP	2	10	0.20	[0.03;0.56]
Schwierzeck, V	2	12	0.17	[0.02;0.48]
Tan, X	2	13	0.15	[0.02;0.45]
Brandstetter, S	2	36	0.06	[0.01;0.19]
Melgosa, M	3	16	0.19	[0.04;0.46]
Wu, HP	3	23	0.13	[0.03;0.34]
Arima, Y	4	12	0.33	[0.10;0.65]
Zhang, W2	4	12	0.33	[0.10;0.65]
Arons, MM	3	47	0.06	[0.01;0.18]
Cheng, HY	4	22	0.18	[0.05;0.40]
Gao, Y	6	15	0.40	[0.16;0.68]
Merza, MA	6	15	0.40	[0.16;0.68]
Wang, Z	4	47	0.09	[0.02;0.20]
Yongchen, Z	5	21	0.24	[0.08;0.47]
Park, SY	4	97	0.04	[0.01;0.10]
Song, W	8	16	0.50	[0.25;0.75]
Dora, AV	6	19	0.32	[0.13;0.57]
Rivett, L	5	30	0.17	[0.06;0.35]
Qiu, C	5	104	0.05	[0.02;0.11]
Tian, S	7	24	0.29	[0.13;0.51]
Bai, K	8	25	0.32	[0.15;0.54]
Zhou, R	9	31	0.29	[0.14;0.48]
Xu, H	11	32	0.34	[0.19;0.53]
Qiu, H	10	36	0.28	[0.14;0.45]
Wu, J	8	83	0.10	[0.04;0.18]
Luo, L	8	129	0.06	[0.03;0.12]
Ma, Y	11	47	0.23	[0.12;0.38]
Kim, SE	10	71	0.14	[0.07;0.24]
Treibel, TA	12	44	0.27	[0.15;0.43]
Solbach, W	10	97	0.10	[0.05;0.18]
Chang, MC	10	139	0.07	[0.04;0.13]
Zhou, X	10	328	0.03	[0.01;0.06]
Angelo Vaira, L	10	345	0.03	[0.01;0.05]
Choe, PG	15	113	0.13	[0.08;0.21]
Bi, Q	17	87	0.20	[0.12;0.29]
Wang, X	14	1012	0.01	[0.01;0.02]
Wong, J	16	138	0.12	[0.07;0.18]
Xu, T	15	342	0.04	[0.02;0.07]
London, V	22	68	0.32	[0.22;0.45]
Sharma, AK	215	234	0.92	[0.88;0.95]
Lavezzo, E	29	73	0.40	[0.28;0.52]
Yang, R	33	78	0.42	[0.31;0.54]
Zhang, W3	26	137	0.19	[0.13;0.27]
Lu, Y	29	110	0.26	[0.18;0.36]
Tabata, S	33	104	0.32	[0.23;0.42]
Lombardi, A	41	138	0.30	[0.22;0.38]
Graham, N	46	126	0.37	[0.28;0.46]
Alshami, AA	69	128	0.54	[0.45;0.63]
Andrikopoulou, M	46	158	0.29	[0.22;0.37]
Noh, JY	53	199	0.27	[0.21;0.33]
Kong, W	45	473	0.10	[0.07;0.13]
Wang, Y2	63	279	0.23	[0.18;0.28]
Pham, TQ	89	208	0.43	[0.36;0.50]
Kumar, R	108	231	0.47	[0.40;0.53]
**Overall effect**		**6616**	**0.2**	**[0.17;0.25]**

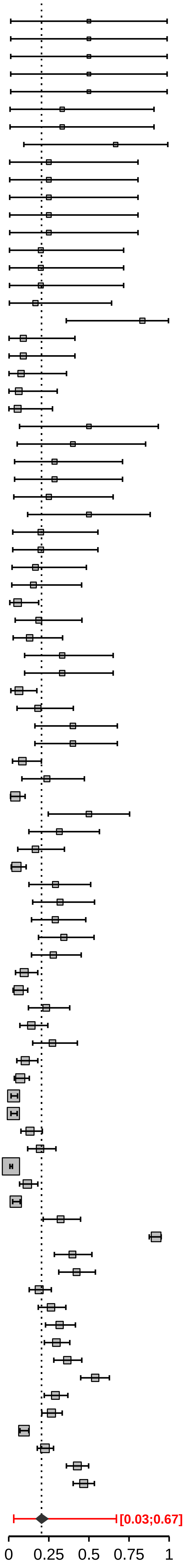

S2 Figure. Review question 1, forest plot of included studies, by study precision

The size of the shaded square is proportional to study size; solid diamond is the summary proportion and 95% confidence interval, estimated from random effects meta-analysis; red line is the prediction interval.
