# Supplementary material for: Occurrence and transmission potential of asymptomatic and presymptomatic SARS-CoV-2 infections: A living systematic review and meta-analysis
Source: PLoS Med. 2020 Sep 22;17(9):e1003346. doi: 10.1371/journal.pmed.1003346 (PMC7508369; doi:10.1371/journal.pmed.1003346)
Supplement: S3 Fig — (PDF) [file pmed.1003346.s005.pdf]

S3 Figure. Risk of bias and reporting quality of observational studies assessing proportion of asymptomatic and pre-symptomatic SARS-CoV-2 infection [11,12]

Author	1 Clear criteria for inclusion	2 RT-PCR diagnosis for all participants	3 Asymptomatic status at start of follow-up	4 Consecutive inclusion of participants	5 Complete inclusion of participants	6 Reporting of the demographics of participants	7 Reporting of clinical information (symptoms)	8a Symptom status of cases at the end of follow-up	8b Adequate follow-up	9 Reporting of the site/clinic demographics	10 Numerator and denominator available	11a Study population adequate sample of the source population	11b Study population adequate sample of the target population	12 The assessment of the secondary attack rate was adequate and similar in all groups (low risk of
**Contact investigations**														
Tong, ZD	Yes	Yes	Yes	Yes	Yes	Yes	Yes	Yes	Yes	Yes	Yes	Yes	No	NA
735: Huang, R	Yes	Yes	Yes	Yes	Yes	Yes	Yes	Yes	Yes	Yes	Yes	Yes	No	NA
344: Jiang, XL	Yes	Yes	Yes	Yes	Yes	Yes	Yes	Yes	Yes	Yes	Yes	Yes	No	NA
372: Jiang, X	Yes	Yes	Yes	Yes	Yes	Yes	No	Yes	Yes	Yes	Yes	Yes	No	NA
36: Liao, J	Yes	Yes	Yes	Yes	Yes	Yes	Yes	Yes	Yes	Yes	Yes	Yes	No	NA
22: Hu, Z	Yes	Yes	Yes	Yes	Yes	Yes	Yes	Yes	Yes	Yes	Yes	Yes	No	NA
42: Luo, SH	Yes	Yes	Yes	Yes	No	No	Yes	Yes	Yes	Yes	Yes	Yes	No	NA
11: Chan, JF	Yes	Yes	Yes	Yes	Yes	Yes	Yes	Yes	Yes	Yes	Yes	Yes	No	NA
180: Ye, F	Yes	Yes	Yes	Yes	Yes	Yes	Yes	Yes	Yes	Yes	Yes	Yes	No	NA
4: Bai, Y	Yes	Yes	Yes	Yes	Yes	No	Yes	Yes	Yes	Yes	Yes	Yes	No	NA
374: Luo: Y	Yes	Yes	Yes	Yes	Yes	No	Yes	Yes	Yes	Yes	Yes	Yes	No	NA
142: Zhang, J	Yes	Yes	Yes	Yes	Yes	No	Yes	Yes	Yes	Yes	Yes	Yes	No	NA
389: Zhang, B	Yes	Yes	Yes	Yes	Yes	Yes	Yes	Yes	Yes	Yes	Yes	Yes	No	NA
449: Huang, L	Yes	Yes	Yes	Yes	Yes	Yes	Yes	Yes	Yes	Yes	Yes	Yes	No	NA
54: Qian, G	Yes	Yes	Yes	Yes	Yes	No	Yes	Yes	Yes	No	Yes	Yes	No	NA
414: Gao, Y	Yes	Yes	Yes	Yes	Yes	Yes	No	Yes	Yes	Yes	Yes	Yes	No	NA
563: Chaw, L	Yes	Yes	Yes	Yes	Yes	Yes	Yes	Yes	Yes	Yes	Yes	Yes	No	Yes
**Contact investigations, aggregated**														
599: Hijen, P	Yes	Yes	Yes	Yes	No	Yes	Yes	Yes	Yes	Yes	Yes	Yes	No	NA
665: Brandstetter, S	Yes	Yes	Yes	Yes	Yes	No	Yes	Yes	Yes	Yes	Yes	Yes	No	NA
821: Zhang, W2	Yes	Yes	Yes	Yes	Yes	Yes	No	Yes	Yes	Yes	Yes	Yes	Yes	Yes
477: Cheng, HY	Yes	Yes	Yes	Yes	Yes	No	No	Yes	Yes	Yes	Yes	Yes	Yes	Yes
249: Wang, Z	No	Yes	Yes	Unclear	No	No	Yes	Unclear	Unclear	Yes	Yes	Unclear	No	NA
597: Wu, J	Yes	Yes	Yes	Yes	Yes	No	Yes	Yes	Yes	Yes	Yes	Yes	No	NA
122: Luo, L	Yes	Yes	Yes	Yes	Yes	Yes	Yes	Yes	Yes	Yes	Yes	Yes	Yes	Yes
443: Bi, Q	Yes	Yes	Yes	Yes	No	No	Yes	Yes	Yes	Yes	Yes	Yes	Yes	NA
908: Yang, R	Yes	Yes	Yes	Yes	Yes	Yes	No	Yes	Yes	Yes	Yes	Yes	No	NA
**Outbreak investigations**														
242: Danis, K	Yes	Yes	Yes	Yes	Yes	No	Yes	Yes	Yes	Yes	Yes	Yes	No	NA
713: Böhmer, MM	Yes	Yes	Yes	Yes	Yes	No	Yes	Yes	Yes	Yes	Yes	Yes	Unclear	NA
763: Roxby, AC	Yes	Yes	Yes	Yes	No	No	Yes	Yes	Yes	Yes	Yes	Yes	No	NA
152: Yang, N	Yes	Yes	Yes	Yes	No	Yes	Yes	Yes	Yes	Yes	Yes	No	No	NA
396: Schwierzeck, V	Yes	Yes	Yes	Yes	Yes	No	Yes	Yes	Yes	Yes	Yes	Yes	No	NA
376: Arons, MM	Yes	Yes	Yes	Yes	Yes	No	No	Yes	Yes	Yes	Yes	Yes	No	NA
354: Park, SY	Yes	Yes	Yes	Yes	Yes	No	No	Yes	Yes	Yes	Yes	Yes	Yes	Yes
849: Dora, AV	Yes	Yes	Yes	Yes	Yes	Yes	Yes	Yes	Yes	Yes	Yes	Yes	No	NA
265: Tian, S	Yes	Yes	Yes	Yes	Yes	Yes	Yes	Yes	Yes	Yes	Yes	Yes	Yes	NA
899: Solbach, W	Yes	Yes	Yes	Yes	No	No	No	Yes	Yes	Yes	Yes	Yes	Yes	NA
1003: Graham, N	Yes	Yes	Yes	Yes	Yes	No	Yes	Yes	Yes	Yes	Yes	Yes	No	NA
696: Pham, TQ	Yes	Yes	Yes	Yes	Yes	No	No	Yes	Yes	Yes	Yes	Yes	Yes	NA
**Screening**														
317: Hoehl, S	Yes	Yes	Yes	Yes	Yes	No	No	Yes	Yes	Yes	Yes	Yes	No	NA
170: Chang, L	Yes	Yes	Yes	Yes	Yes	No	No	Yes	Yes	No	Yes	Yes	No	NA
224: Arima, Y	Yes	Yes	Yes	Yes	Yes	No	No	Yes	Yes	Yes	Yes	No	No	NA
598: Rivett, L	Yes	Yes	Yes	Yes	Yes	No	No	Yes	Yes	Yes	Yes	Yes	No	NA
622: Treibel, TA	Yes	Yes	Yes	Yes	No	No	No	Yes	Yes	No	Yes	Unclear	No	NA
294: Lavezzo, E	Unclear	Yes	Yes	Unclear	No	No	No	Yes	Yes	Yes	Yes	Yes	Unclear	NA
593: Lombardi, A	Yes	Yes	Yes	Yes	Yes	No	Yes	Yes	Yes	Yes	Yes	Yes	No	NA
278: Lytras, T	Yes	Yes	No	Yes	Yes	No	Yes	Yes	Yes	Yes	Yes	Yes	No	NA
**Hospitalised adults**														
204: Pongpirul, WA	Yes	Yes	Yes	Yes	Yes	Yes	Yes	Yes	Yes	Yes	Yes	Yes	No	NA
216: Zou, L	Yes	Yes	Yes	Yes	Unclear	Yes	Yes	Yes	Yes	Yes	Yes	Unclear	No	NA
521: Qiu, C	Yes	Yes	Yes	Yes	Yes	Yes	Yes	Yes	Yes	Yes	Yes	Yes	No	NA
761: Zhou, R	Yes	Yes	Yes	Yes	Yes	No	No	Yes	Yes	Yes	Yes	Unclear	No	NA
664: Chang, MC	Yes	Yes	Yes	Yes	Yes	Yes	No	Yes	Yes	Yes	Yes	Yes	No	NA
233: Zhou, X	Yes	Yes	Yes	Yes	Yes	Yes	Yes	Yes	Yes	Yes	Yes	Yes	No	NA
765: Angelo Vaira, L	Yes	Unclear	Yes	Yes	Yes	No	Yes	Yes	Yes	Yes	Yes	Yes	No	NA
187: Wang, X	Yes	Yes	Yes	Yes	Yes	Yes	Yes	Yes	Yes	Yes	Yes	Yes	No	NA
506: Wong, J	Yes	Yes	Yes	Yes	Yes	No	No	Yes	Yes	No	Yes	Yes	No	NA
434: Xu, T	Yes	Yes	Yes	Yes	Yes	Yes	Yes	Yes	Yes	Yes	Yes	Yes	No	NA
729: London, V	Yes	Yes	Yes	Yes	Unclear	Yes	No	Yes	Yes	Yes	Yes	Yes	No	NA
164: Tabata, S	Yes	Yes	Yes	Yes	Yes	Yes	Yes	Yes	No	Yes	Yes	No	No	NA
832: Andrikopoulou, M	Yes	Yes	Yes	Yes	Yes	No	Yes	Yes	Yes	Yes	Yes	Yes	No	NA
797: Noh, JY	Unclear	Yes	Yes	Unclear	Unclear	No	Yes	Yes	Yes	Yes	Yes	Yes	Unclear	NA
818: Kumar, R	Yes	Yes	Yes	Yes	No	Yes	Yes	Yes	Yes	Yes	Yes	Yes	No	NA
267: Meng, H	Yes	Yes	Yes	Yes	Unclear	Yes	Yes	Yes	Yes	Yes	Yes	Yes	No	NA
491: Zhang, Z	Yes	Yes	Yes	Yes	No	Yes	No	Yes	Yes	Yes	Yes	Yes	No	NA
834: Al-Shamsi, HO	Yes	Yes	Yes	Yes	Yes	Yes	No	Yes	Yes	Yes	Yes	Yes	No	NA
71: Wang, Y1	Yes	Yes	Yes	Yes	Yes	Yes	No	No	Yes	Yes	Yes	Yes	No	NA
**Hospitalised children**														
299: See, KC	Yes	Yes	Yes	Yes	Yes	Yes	Yes	Yes	Yes	Yes	Yes	Unclear	No	NA
275: Tan, YP	Yes	Yes	Yes	Yes	Yes	Yes	Yes	Yes	Yes	Yes	Yes	Yes	No	NA
334: Tan, X	Yes	Yes	Yes	Yes	Yes	Yes	Yes	Yes	Yes	Yes	Yes	Yes	No	NA
769: Melgosa, M	Yes	Yes	Yes	Yes	Yes	No	Yes	Yes	Yes	Yes	Yes	Yes	No	NA
843: Wu, HP	Yes	Yes	Yes	Yes	Yes	No	Yes	Yes	Yes	Yes	Yes	Yes	No	NA
481: Song, W	Yes	Yes	Yes	Yes	Unclear	Yes	Yes	Yes	Yes	Yes	Yes	Yes	No	NA
653: Bai, K	Yes	Yes	Yes	Yes	Yes	No	Yes	Yes	Yes	Yes	Yes	Yes	No	NA
382: Xu, H	Yes	Yes	Yes	Yes	Unclear	Yes	Yes	Yes	Yes	Unclear	Yes	Yes	No	NA
141: Qiu, H	Yes	Yes	Yes	Yes	Yes	Yes	Yes	Unclear	Unclear	Yes	Yes	Yes	No	NA
556: Lu, Y	Yes	Yes	Yes	Yes	Unclear	Yes	Yes	Yes	Yes	Yes	Yes	Yes	No	NA
**Hospitalised adults and children**														
637: Merza, MA	Yes	Yes	Yes	Yes	Yes	Yes	Yes	Yes	Yes	Yes	Yes	Yes	No	NA
328: Yongchen, Z	Yes	Yes	Yes	Yes	Yes	Yes	No	Yes	Yes	Yes	Yes	Yes	No	NA
585: Ma, Y	Yes	Yes	Yes	Yes	Yes	Yes	Yes	Yes	Yes	Yes	Yes	Yes	No	NA
559: Kim, SE	Yes	Yes	Yes	Yes	Yes	Yes	Yes	Yes	Yes	Yes	Yes	Yes	Unclear	NA
856: Choe, PG	Yes	Yes	Yes	Yes	No	Yes	Yes	Yes	Yes	Yes	Yes	No	No	NA
981: Sharma, AK	Yes	Yes	Yes	Yes	Yes	No	No	Yes	Yes	Yes	Yes	Yes	No	NA
745: Zhang, W3	Unclear	Yes	Yes	Yes	No	No	Yes	Yes	Yes	Yes	Yes	Unclear	No	NA
726: Alshami, AA	Yes	Yes	Yes	Yes	Yes	No	Yes	Yes	Yes	Yes	Yes	Yes	Unclear	NA
936: Kong, W	Yes	Yes	Yes	Yes	No	Yes	Yes	Yes	Yes	Yes	Yes	Yes	No	NA
778: Wang, Y2	Yes	Yes	Yes	Yes	Yes	Yes	Unclear	Yes	Yes	Yes	Yes	Yes	No	NA

Adapted from two published assessment tools, citations refer to reference list in main text.
11. Joanna Briggs Institute checklist for case series, questions 1 to 10 (http://joannabriggs.org/research/critical-appraisal-tools.html)
12. Boyle MH. Guidelines for evaluating prevalence studies. Evidence Based Men Health 1998;1:37-40, questions 11a and 11b
Question 12 added by authors to assess risk of selection bias for pariticipants in studies reporting secondary attack rate
