# Supplementary figures and images for: Occurrence and transmission potential of asymptomatic and presymptomatic SARS-CoV-2 infections: A living systematic review and meta-analysis
Source: PLoS Med. 2020 Sep 22;17(9):e1003346. doi: 10.1371/journal.pmed.1003346 (PMC7508369; doi:10.1371/journal.pmed.1003346)

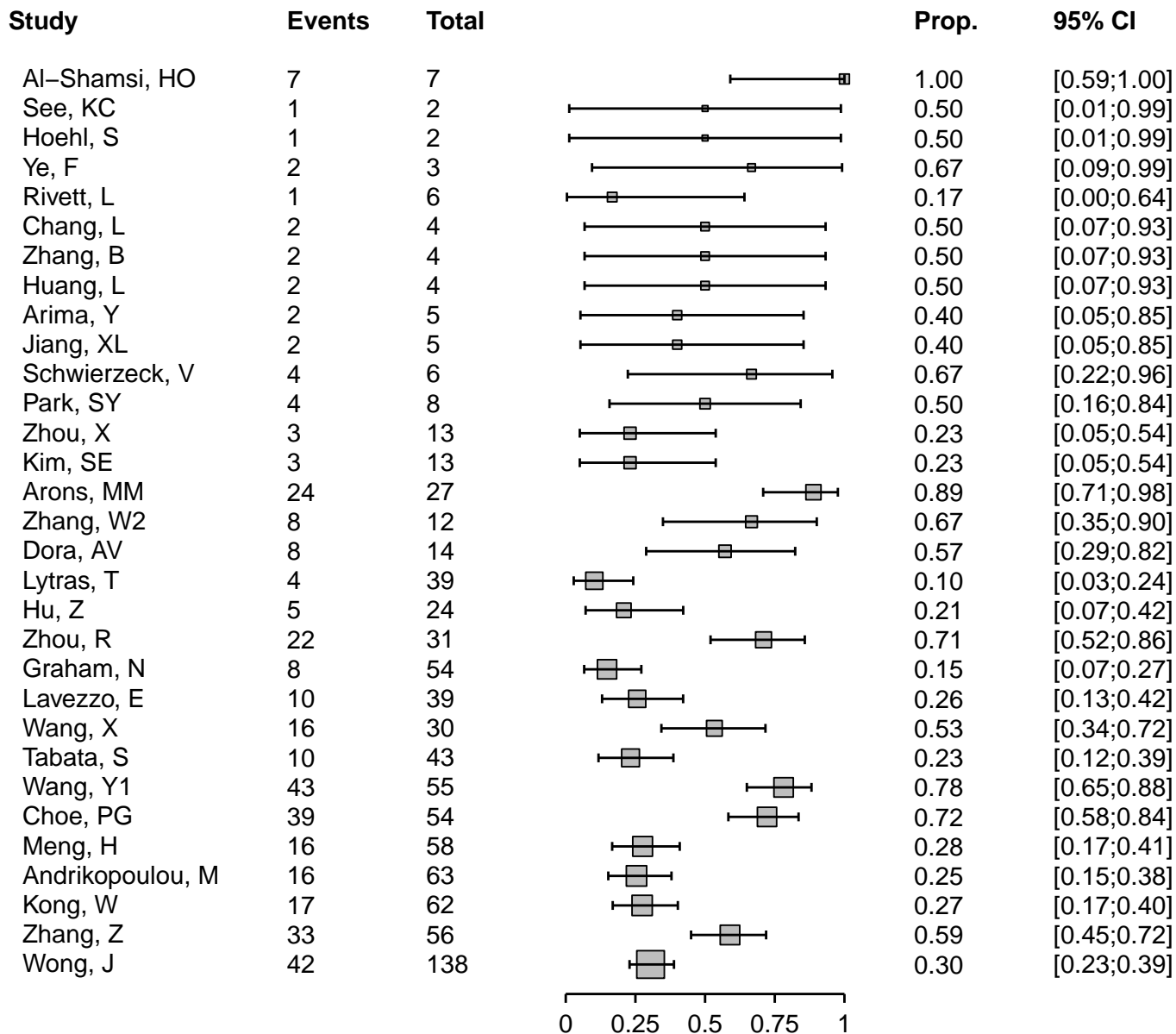

**S4 Figure: Review question 2, forest plot of included studies, by study precision**

Supplement: S4 Fig — (PDF) [file pmed.1003346.s006.pdf]
