# Supplementary material for: Occurrence and transmission potential of asymptomatic and presymptomatic SARS-CoV-2 infections: A living systematic review and meta-analysis
Source: PLoS Med. 2020 Sep 22;17(9):e1003346. doi: 10.1371/journal.pmed.1003346 (PMC7508369; doi:10.1371/journal.pmed.1003346)
Supplement: S7 Fig — (PDF) [file pmed.1003346.s009.pdf]

S7 Figure. Relevance and credibility of mathematical modelling studies assessing contribution of asymptomatic and pre-symptomatic infection to SARS-CoV-2 transmission

Author	1	2	3	4	5	6	7	8	9	10	11	12	13
	Is the population relevant?	Are any critical interventions missing?	Is the context (settings and circumstances) applicable?	Is external validation of the model sufficient to make its results credible for your decision?	Is internal verification of the model sufficient to make its results credible for your decision?	Does the model have sufficient face validity to make its results credible for your decision?	Is the design of the model adequate for your decision problem?	Are the data used in populating the model suitable for your decision problem?	Were the analyses performed using the model adequate to inform your decision problem?	Was there an adequate assessment of the effects of uncertainty?	Was the reporting of the model adequate to inform your decision problem?	Was the interpretation of results fair and balanced?	Were there any potential conflicts of interest?
Ferretti, L	Partial	NA	Yes	No	Yes	Yes	Yes	Partial	Yes	Yes	Yes	No	No
Ganyani, T	Partial	NA	Yes	No	Yes	Yes	Yes	Partial	Yes	Partial	Yes	Partial	No
Zhang, W	Partial	NA	Yes	No	Yes	No	Partial	Partial	No	No	No	No	Unclear
He, X	Partial	NA	Yes	No	Yes	Yes	Yes	Partial	Yes	Yes	Partial	Yes	No
Emery, JC	Partial	NA	Yes	Partial	Yes	Yes	Partial	Partial	Yes	Yes	Yes	Yes	No
Casey, M	Yes	NA	Yes	Partial	Yes	Partial	Yes	Yes	Partial	Yes	Yes	Yes	No
Kim, Y	Partial	NA	Yes	No	Yes	Yes	Yes	Partial	Yes	Yes	Yes	Partial	No
Peak, CM	Yes	NA	Partial	No	Yes	Partial	Yes	No	Partial	Partial	Partial	Yes	No

Checklist from ref 13. Jaime Caro J, et al. Value Health 2014;17:174-82.
Partial: item is partially addressed
NA: Not applicable
