# Supplementary material for: Occurrence and transmission potential of asymptomatic and presymptomatic SARS-CoV-2 infections: A living systematic review and meta-analysis
Source: PLoS Med. 2020 Sep 22;17(9):e1003346. doi: 10.1371/journal.pmed.1003346 (PMC7508369; doi:10.1371/journal.pmed.1003346)
Supplement: S1 Table — (DOCX) [file pmed.1003346.s010.docx]

**S1 Table. Changes over time in types of evidence about asymptomatic SARS-CoV-2 infection. Number of new studies identified, by date of search**

| **Version** | **Version 1** | **Version 2** | **Version 3** | **Overall** |
| --- | --- | --- | --- | --- |
| **Search date** | **25.03.2020** | **20.04.2020** | **10.06.2020** |  |
| Observational studies |  |  |  |  |
| Contact investigation | 6 | 4^a^ | 7 | 17^a^ |
| Evacuation | 2^b^ | .. | .. | .. |
| Contact investigation, aggregated | .. | 2 | 7^c^ | 9^c^ |
| Outbreak investigation | .. | 4^c^ | 8 | 12 |
| Screening | .. | 4^b^ | 4 | 8^b^ |
| Hospitalised adults | .. | 8^d^ | 10 | 18^d^ |
| Hospitalised children | .. | 3 | 7 | 10 |
| Hospitalised adults and children | .. | .. | 10 | 10 |
| Secondary attack rate^e^ | .. | .. | 1^e^ | 1 |
| Statistical modelling | 1 | 0 | 0 | 1 |
| Mathematical modelling | 2 | 2 | 4 | 8 |
| **Total** | **11** | **25^b^** | **58** | **94** |

Footnotes refer to specific studies, where data from one version have been updated in a new publication. Citations use the number of the study in the reference list in the main text.

1. In version 3, one study (Le TQM [30]) does not appear in forest plot because participants were included in a larger study (Wong J [103]), which was categorised with hospitalised adults)
2. In version 2, two studies of evacuees were re-distributed into new categories: screening (Arima Y [28], which replaces Nishiura H [25]) and hospitalised adults (Tabata S [27]). In the ‘Overall’ column total, these studies are included in their new categories. In the column total for version 2, these studies are not included because they are already counted in version 1;
3. In version 3, one study (Kimball A [35]) replaced (by Arons M [58]) with updated data
4. In version 3, one study (Breslin N [29]) replaced (by Andrikopoulou [56]) with updated data
5. New category in version 3. Chaw L [65] contributes data only to calculation of secondary attack rate. Four studies in other categories also contribute to this analysis
