# Supplementary material for: Occurrence and transmission potential of asymptomatic and presymptomatic SARS-CoV-2 infections: A living systematic review and meta-analysis
Source: PLoS Med. 2020 Sep 22;17(9):e1003346. doi: 10.1371/journal.pmed.1003346 (PMC7508369; doi:10.1371/journal.pmed.1003346)
Supplement: S2 Table — (DOCX) [file pmed.1003346.s011.docx]

**S2 Table. Country in which studies were done to estimate proportions of asymptomatic and pre-symptomatic SARS-CoV-2 infections**

| **Review question 1, proportion of asymptomatic infection** | | |
| --- | --- | --- |
| **Country** | **Number of studies**^a^ | **SARS-CoV-2 cases, total** |
| China | 44 | 3619 |
| Germany | 6 | 174 |
| South Korea | 5 | 619 |
| USA | 5 | 298 |
| Italy | 3 | 556 |
| United Kingdom | 3 | 200 |
| India | 2 | 465 |
| Brunei | 1 | 138 |
| Cruise Ship | 1 | 104 |
| France | 1 | 13 |
| Iraq | 1 | 15 |
| Japan | 1 | 12 |
| Malaysia | 1 | 4 |
| Saudi Arabia | 1 | 128 |
| Spain | 1 | 16 |
| Taiwan | 1 | 22 |
| Thailand | 1 | 11 |
| Vietnam | 1 | 208 |
| Total | 79 | 6602 |
|  |  |  |
| **Review question 2: proportion of pre-symptomatic infection** | | |
| **Country** | **Number of studies**^a^ | **Asymptomatic SARS-CoV-2 cases at time of testing, total** |
| China | 14 | 353 |
| South Korea | 3 | 75 |
| USA | 3 | 104 |
| Germany | 2 | 8 |
| Japan | 2 | 48 |
| Brunei | 1 | 138 |
| Greece | 1 | 39 |
| Italy | 1 | 39 |
| Malaysia | 1 | 2 |
| United Arab Emirates | 1 | 7 |
| United Kingdom | 2 | 60 |
| Total | 31 | 873 |

^a^ A study can contribute data to more than one review question.
