# Supplementary material for: Occurrence and transmission potential of asymptomatic and presymptomatic SARS-CoV-2 infections: A living systematic review and meta-analysis
Source: PLoS Med. 2020 Sep 22;17(9):e1003346. doi: 10.1371/journal.pmed.1003346 (PMC7508369; doi:10.1371/journal.pmed.1003346)
Supplement: Attachment — Submitted filename: Resonse_200725.pdf [file pmed.1003346.s012.pdf]

ISPM – Mittelstrasse 43, CH-3012 Bern

b UNIVERSITÄT BERN

Faculty of Medicine

Institute of Social and Preventive Medicine

Requests from the editors:

1. *As one referee suggests, please update the search.*

We anticipate the need to update the search and we did this on 10th June. We screened 688 new records and now include a total of 94 studies.

2. *Please state in some additional detail how you plan to maintain the "living" status of the review.*

We have added additional details to a version 4.0 of our protocol (https://osf.io/9ewys/) and summarise in the Methods, Information and sources (lines 125 and 134-135), "We conducted the first search on March 25, 2020 and updated it on April 20 and June 10, 2020… Reports from this living rapid systematic review will be updated at three months intervals, with continuously updated searches." text of the review,…

We have also replied to Reviewer #1's comments about other aspects of the living systematic review in detail.

3. *Please remove the word "rapid" from the title. We suggest adapting the title to: "Development and transmission of asymptomatic SARS-CoV-2 infections: a living systematic review and meta-analysis".*

We have removed 'rapid' from the title. Could we suggest, "Asymptomatic SARS-CoV-2 infections: a living systematic review and meta-analysis" The term 'Development… of asymptomatic infection' isn't really accurate because asymptomatic status is the starting point.

4. *Please add a new final sentence to the "methods and findings" subsection of your abstract, quoting 2-3 of your study's main limitations.*

We have added to the Abstract (lines 45-49), "Limitations of the review include that most included studies were not designed to estimate the proportion of asymptomatic SARS-CoV-2 infections and were at risk of selection biases, we did not consider the possible impact of false negative RT-PCR results, which would underestimate the proportion of asymptomatic infections, and that the database does not include all sources."

5. *We suggest reversing the order of the two sentences making up the "conclusions" subsection of your abstract.*

We have reversed the sentences. The Conclusion (lines 51-55) now reads, "The findings of this living systematic review of publications early in the pandemic suggest that most SARS-CoV-2 infections are not asymptomatic throughout the course of infection. An intermediate contribution of pre-symptomatic and asymptomatic infections to overall SARS-CoV-2 transmission means that combination prevention measures, with enhanced hand and respiratory hygiene, testing tracing and isolation strategies and social distancing, will continue to be needed. "

Prof. Dr. Nicola Low
Professor of Epidemiology and Public Health
Institute of Social and Preventive Medicine
Mittelstrasse 43
CH-3012 Bern

Tel. +41 031 631 30 92
nicola.low@ispm.unibe.ch
www.ispm.unibe.ch

6. *After the abstract, we will need to ask you to add a new and accessible "author summary" section in non-identical prose. You may find it helpful to consult one or two recent research papers in PLOS Medicine to get a sense of the preferred style.*

We have written an author summary, following the editorial instructions (lines 56-84).

7. *You may wish to briefly explain what a "shiny app" is.*

We apologise for the jargon. We have rephrased this (line 314) to explain that it is an 'interactive web application …

8. *We would be interested to know whether you see any potential issues in including data from preprints. Would it be possible to report sensitivity analyses omitting these data?*

This is an interesting question. We have added some more information about preprints (lines 203-205), "At the time of their inclusion in the review, 23 of the included records were preprints; six of these had been published in peer-reviewed journals by 17 July 2020 [19,20,27,81,82,106]." We have done a sensitivity analysis for the estimated proportion of asymptomatic infections, which we include as supporting information (S6 Figure). We report in the text (lines 292-295). "To examine publication status, we conducted a sensitivity analysis, omitting studies that were identified as preprints at the time of data extraction (S6 Figure). The estimate of the proportion of asymptomatic infection in all settings (18%, 95% CI 14–22%) and setting-specific estimates were very similar to the main analysis."

9. *Throughout the text, please add p values alongside 95% CI, where available.*

Most of our results are proportions with 95% CI, so no p-values are necessary. We have summarised all subgroup and sensitivity analyses in a single section now (Additional analyses, lines 285-295). Since these comparisons are exploratory, they do not require statistical hypothesis tests.

10. *Please adapt reference call outs so that they precede punctuation, e.g. "... a single point [2,3]." (i.e., removing spaces within the square brackets).*

Apologies. We have corrected the formatting errors.

11. *Is reference 1 lacking a report number?*

Apologies. This is an automated field in Endnote. The report doesn't have a number. We have deleted the field.

12. *Please add the journal details to reference 4.*

We have deleted this reference from the revised version.

13. *Where you list preprints in your reference list, e.g., references 25 and 26, please add "[preprint]".*

We now indicate “[preprint]” in all citations, where relevant.

14. *In the attached PRISMA checklist, please refer to individual items by section (e.g., "Methods") and paragraph number rather than by line or page numbers, as the latter generally change in the event of publication.*

We have adapted the PRISMA checklist, as requested.

Comments from the reviewers:

***** Reviewer #1:***

1. *First, it is claimed in multiple places that this is a"living review" but this is not a living review according to common definitions (including that used by Elliott et al in Plos Medicine). Rather, it appears that the authors created a database that merges 4 other databases and searched it twice. The review itself is important and credible, and there is no need to claim that it is something that it is not (ie living).*

We are grateful for the opportunity to clarify why our study is a “living systematic review”, according to Elliott and colleagues’ definition in PLOS Medicine (2014;11(2):e1001603, cited as ref 7), “Living systematic reviews are high quality, up-to-date online summaries of health research, updated as new research becomes available, and enabled by improved production efficiency and adherence to the norms of scholarly communication.” We use automated workflow methods that the Living Systematic Review Network recommends (Thomas J, et al. J Clin Epidemiol 2017;91:31-37, cited as ref 8). We have made the following changes in the revised version to describe the methods and outputs of the living systematic review:

Methods (lines 111-115), “We conducted a living systematic review, a systematic review that provides an online summary of findings and is updated when relevant new evidence becomes available [7]. The review follows a published protocol (https://osf.io/9ewys/), which describes in detail the methods used to speed up review tasks [8]. The first two versions of the review have been published as preprints [10, 11].”

Methods, Information sources and searches (lines 125-135), “We conducted the first search on March 25, 2020 and updated it on April 20 and June 10, 2020. We searched the covid-19 living evidence database [13], which is generated using automated workflow processes [8] to: i) provide daily updates of searches of four electronic databases: Medline Pubmed, Ovid Embase, bioRxiv and medRxiv, using medical subject headings and free text keywords for SARS-CoV-2 infection and covid-19; ii) de-duplicate the records; iii) tag records that are preprints; and iv) allow searches of

titles and abstracts using Boolean operators. We used the search function to identify studies of asymptomatic or pre-symptomatic SARS-CoV-2 infection using a search string of medical subject headings and free text keywords (Data supplement, S1 Text). We also examined articles suggested by experts and the reference lists of retrieved mathematical modelling studies and systematic reviews. Reports from this living rapid systematic review will be updated at three months intervals, with continuously updated searches."

Results (lines 193-201), "In the first version of the review [7], 11 articles were eligible for inclusion [17-27], version 2 [8] identified another 26 eligible records [28-53], and version 3 identified another 61 eligible records [54-114]. After excluding four articles for which more recent data became available in a subsequent version [25,29,30,35], the total number of articles included was 94 (S1 Table) [17-24,26-28,31-34,36-114]. The types of evidence changed across the three versions of the review (S1 Table). In the first version, six of 11 studies were contact investigations of single family clusters with a total of 39 people. In the next versions, study designs included larger investigations of contacts and outbreaks, screening of defined groups and studies of hospitalised adults and children."

2. *Second, living or not, it would be important to update the search prior to publication. I appreciate that this may require considerable work in terms of data extraction and analysis; however, the last search was conducted in mid April and a number of studies have been published since that time.*

We agree. Indeed, we updated our search on 10 June 2020, resulting in 688 new records for screening and the inclusion of 58 additional records for a total of 94 included studies (S1 Figure, Flow chart).

Specific comments

3. *Line 35: "using a living evidence database of SARS-CoV-2 literature" What does this mean*

We describe this in the Abstract now (line 30-31) as "a database of SARS-CoV-2 literature that is updated daily" The description in the main text (lines 125-135) is in our response to comment 1.

4. *Line 40: Risk of bias was assessed using a questionnaire for modelling studies? Use of a* questionnaire to assess bias is unclear? (It is clearer in the text)

We have amended this sentence (lines 35-36) to, "Risk of bias in empirical studies was assessed with an adapted checklist for case series and the relevance and credibility of modelling studies were assessed using a published checklist."

5. *Line 61: Substantial disagreement' is claimed by contrasting quotes from a report in mid February and a news article in April. This is journalism and does not belong in a medical journal.*

We have revised the text with more recent examples from a narrative review by Oran DP and Topol EJ (cited as ref 1) (lines 85-89), “There is ongoing discussion about the level of asymptomatic severe acute respiratory syndrome coronavirus 2 (SARS-CoV-2) infection. The authors of a narrative review report a range of proportions of participants positive for SARS-CoV-2 but asymptomatic in different studies from 6 to 96% [1].”

6. *Line 84: There are multiple places where this review is claimed to be a 'living' review but no definition is given and it is unclear what makes this review 'living' other than the fact that the search has been updated at least once (which is common for most reviews).*

Please see our response to comment 1.

7. Line 95: "We searched the covid-19 living evidence database…"

- If I understand correctly, this is a single database that combines records from 4 databases. To me this doesn't fullfil the definition of a "living evidence database" (the same could otherwise be said just about PubMed), and having searched it twice doesn't make this a 'living systematic review'.

Please see our reply to comment 1.

****** Reviewer #2:***

While I have no important concerns about the methods or conclusions, I have a few minor comments. I also wonder if the authors could address in the discussion section a few additional questions that are pertinent:

1. *Are there relationships among inoculum dose, peak viral load, and likelihood of symptoms? It seems there's a relationship between viral load and infectiousness -- but do we know about this and symptoms? In people or, if no human data, in the macaque infection model?*

Thank you. We agree that this the biological basis for asymptomatic infection is important. In the interests of space we have only been able to address these points briefly in the Discussion (lines 410-415), “Integration of evidence from epidemiological, clinical and laboratory studies will help to clarify the relative infectiousness of asymptomatic SARS-CoV-2. Studies using viral culture as well as RNA detection are needed since RT-PCR defined viral loads appear to be broadly similar in asymptomatic and symptomatic people [136,137]. Age might play a role as children appear more likely than adults to have an asymptomatic course of infection (Figure 1) [138]; age was poorly reported in studies included in this review (Table 1).”

2. *Is there evidence to support that the likelihood of symptomatic infection depends on age, comorbidities, or other demographic factors?*

In addition to the Discussion, we examined information about age. We have now stratified our results in Figure 1, to show, where available, estimates separately for children and adults and (lines 286-288), “In studies of hospitalised children, the point estimate was higher (25%, 95% CI

14–40%, 10 studies) than in adults (11%, 95% CI 7–17%, 15 studies), but confidence intervals overlapped (Figure 1). ”

3. *Even if we don't have compelling data for these, to what extent might they play a role in the central topics of this manuscript? It might be good to point out in the discussion section types of research beyond the ones reviewed that might inform the manuscript's main topics.*

We hope that the inclusion of the clinical laboratory studies mentioned above addresses this important comment.

Minor points:

Abstract

4. *Line 47. Might be worth clarifying that the inference that 40-60% of infections from presymptomatic transmission comes from modeling studies fit to data?*

We agree. The evidence from modelling studies has changed so the sentence now reads (lines 43-44), “Modelling studies fit to data found a higher proportion of all SARS-CoV-2 infections resulting from transmission from pre-symptomatic individuals than from asymptomatic individuals.”

5. *Line 50. I'm not sure what 'intermediate' means, or what it's intermediate between. Is there a way to restate this quantitatively?*

We have deleted this sentence.

Discussion.

6. *In the 'implications and unanswered questions' section, it might be worth caveating many of the statements about various interventions that will be needed for controlling transmission. For example, digital contact tracing may not be necessary for control (though in theory it would be helpful) -- it is still unproven in practice.*

We agree and have revised the sentence to say (lines 422-424), “Digital, proximity tracing could supplement classical contact tracing to speed up detection of contacts to interrupt transmission during the pre-symptomatic phase if shown to be effective [19,127].”

****** Reviewer #3:***
I confine my remarks to statistical aspects of this paper. These were well done and I recommend publication.

****** Reviewer #4:***
1. *L59: the authors reversed that claim more recently, so it’s probably not fair to hold them to it.*

Thank you. We have replaced this statement. Please see our response to Reviewer #1, comment 5.

2. *L74: I'm not sure I agree with this statement. Testing can still be an important part of control if the proportion of asymptomatic infections is high. It's just that you would need to test people who are asymptomatic as well as those who are symptomatic, e.g. through active case finding, drive by test centres for contacts of cases, sweeps of particular groups. In other words, what many Asian countries are doing.*

We agree. We have revised this sentence (line 100-103) to say, "If, however, most transmission is from people without symptoms, social distancing measures that reduce contact with people who might be infectious, should be prioritised, enhanced by active case-finding through testing of asymptomatic people."

3. *Box 1: So someone who says they feel fine but have a fever of 38 degrees measured objectively would not be considered symptomatic? I ask because my colleagues see people who meet those criteria and although formally they may not be 'symptomatic' in the sense of not being patient reported they probably merit being counted as 'symptomatic' in the sense of the infection manifesting an effect on the body.*

We agree that there is a blurred line between subjective symptoms and clinical signs. In clinical practice, we would expect clinical judgement to prevail. We use this definition for published studies to exclude isolated findings such as crackles on auscultation in the absence of patient-reported respiratory symptoms. We have not made any changes to the text in response to this comment.

4. *Box 1: Asymptomatic infection: so how about someone who is positive on serology? That would presumably be a better test of infection since the timing is less of an issue.*

We did not include studies that used serology for diagnosis. Serological studies bring their own challenges, so we now mention this in the Discussion (lines 389-392), "Serological tests, in combination with virological diagnostic methods, might improve ascertainment of SARS-CoV-2 infection in asymptomatic populations. Prospective documentation of symptom status would be required, and improvements in the performance of serological tests are still needed [123]"

5. *L95: Might be good to update prior to publication. Sorry!*

We had anticipated this. We updated our search on 10 June 2020, as we mention in our response to the editors' and reviewer #1.

6. *L128: Presumably other proportions also have uncertainty intervals so why highlight the interval only for proportion of transmission prior to onset*

This was to distinguish uncertainty/credibility intervals from confidence intervals. We agree that is is unnecessary and have deleted this from the study objectives (lines 104-108).

7. L260: missing parentheses

Corrected.

8. L287: are initials required?

We have given the initials of authors because several Chinese authors have the same last name and some have the same initials (for example, we now have Zhang W1 (ref 51), Zhang W2 (ref 111) and Zhang W3 (ref 112)). For consistency, we have also given the first initial of authors with non-Chinese names.

9. L328: missing 'that'; the comma on L329 should be a semi colon

Thank you, we have corrected these typos.

10. L350: this sentence should be clarified

This sentence is no longer in the revised Discussion.

11. L351: surely ALL symptomatic cases are presymptomatic at some point!

We agree. We reworded, and moved, this sentence. It now reads (lines 394-396), "Since all people infected with SARS-CoV-2 are initially asymptomatic, the proportion that will go on to develop symptoms can be derived by subtraction from the estimated proportion with true asymptomatic infections…"

12. F1: perhaps change 'effect' to 'estimate'

We agree and have corrected figures in the revised.
